# Prevalence and molecular characterization of *Toxoplasma gondii* in different types of poultry in Greece, associated risk factors and co-existence with *Eimeria* spp.

**DOI:** 10.1007/s00436-022-07701-6

**Published:** 2022-10-29

**Authors:** Marianna Andreopoulou, Gereon Schares, Martin Koethe, Ilias Chaligiannis, Pavlo Maksimov, Maike Joeres, Garance Cardron, Tina Goroll, Smaro Sotiraki, Arwid Daugschies, Berit Bangoura

**Affiliations:** 1 Institute of Parasitology, Centre for Infectious Diseases, Leipzig University, Leipzig, Germany; 2 Veterinary Research Institute, Hellenic Agricultural Organization ELGO-DIMITRA, 57001 Thessaloniki, Thermi Greece; 3 Friedrich-Loeffler-Institut, Federal Research Institute for Animal Health, National Reference Laboratory for Toxoplasmosis, Greifswald-Insel Riems, Germany; 4 Institute of Food Hygiene, Faculty of Veterinary Medicine, Leipzig University, Leipzig, Germany; 5 Clinic of Farm Animals, Faculty of Veterinary Medicine, School of Health Sciences, Aristotle University of Thessaloniki, 54627 Thessaloniki, Greece; 6 Department of Veterinary Sciences, University of Wyoming, Laramie, WY USA

**Keywords:** Chicken, ELISA, Bioassay, Magnetic capture PCR, Microsatellite typing, Seroprevalence

## Abstract

*Toxoplasma gondii* is a protozoan parasite of public health importance, infecting all warm-blooded animals, including chickens. Undercooked chicken meat or relevant products such as sausages could lead to human infections. In free-range, organic and slow-growth farming systems where the susceptibility period for chickens is extended, more knowledge about potential risk factors is essential. This study is the first seroepidemiological survey in different regions and types of chicken farms in Greece, using a major tachyzoite surface antigen-based ELISA (TgSAG1), combined with magnetic-capture PCR (mc-PCR) and bioassay for the isolation of strains from the chickens’ tissues. Potential risk factors for *T. gondii* infection in these hosts were also investigated. Additionally, the co-existence of *T. gondii* and *Eimeria* spp. infections was assessed to elucidate epidemiological links between these two protozoan infections. Overall *T. gondii* seroprevalence was 9.5%. Of the backyard chickens sampled, 41.2% were seropositive and 70% of the organic and free-range layer farms had at least one *T. gondii* seropositive hen. No serologically positive broilers were found, although mc-PCR revealed a positive sample, highlighting the importance of accurate early-infection direct detection of *T. gondii* infections to ensure public health. *T. gondii* isolates obtained by mouse bioassay were genotyped. All belonged to type II (ToxoDB#3) as confirmed also by microsatellite typing. Production system, type of nutrition, and feeding system automation were identified as the most significant risk factors, while no association was found between the presence of cats and *T. gondii* seropositivity as calculated on both a farm level and per individual bird sampled.

**Supplementary Information:**

The online version contains supplementary material available at 10.1007/s00436-022-07701-6.

## Introduction

Toxoplasmosis is a widespread parasitic zoonosis, caused by the obligate intracellular protozoan parasite *Toxoplasma gondii,* which can infect all warm-blooded animals, i.e., mammals and avian species (Dubey 2010a). Felids are the definitive hosts of *T. gondii* (Montoya and Liesenfeld 2004).

Cats can shed millions of oocysts in their faeces that can survive for months even under outdoor conditions. The oocysts sporulate and become infective within 1–5 days, depending on the climate conditions (Dubey 2009). Birds and mammals, including humans, can be infected either by ingestion of sporulated oocysts from water or unwashed contaminated food that has previously been contaminated with cats’ faeces. Other sources of infection can be the consumption of tissue cysts from raw or undercooked meat (Dubey 2009) or vertical transplacental transmission in mammals (Montoya and Liesenfeld 2004, Sarr et al. 2020).

Chickens, especially free-range chickens, have been hypothesized as important intermediate hosts in the epidemiology of toxoplasmosis. Since chickens have outdoor access, feed from the ground, and are susceptible to infection, this species can act as an indicator of environmental contamination with *T. gondii* (Dubey 2010b; Moré et al. 2012; Hill and Dubey 2013; Dubey et al. 2015;), and as sentinels in regions with a high prevalence of *T. gondii* infection in humans (Dubey et al. 2002). Therefore, free-range chickens have been widely used to study the prevalence and genetic variation of *T. gondii* worldwide (Chikweto et al. 2011; Wang et al. 2013; Verma et al. 2015; Vieira et al. 2018a, b; Rodrigues et al. 2019).

A number of studies suggest that poultry may represent an important source of infection for definitive hosts, like domestic cats but also for humans through the consumption of undercooked infected chicken meat (Tenter et al. 2000; Dubey 2010a, b; Stelzer et al. 2019).

*T. gondii* presents a complex population structure, with three clonal lineages, type I, II, and III prevailing in Europe and North America (Howe and Sibley 1995; Fernández-Escobar et al. 2022). Globally, a number of studies revealed a much greater genetic diversity of the parasite (Khan et al. 2006, 2007; Lehmann et al. 2006; Pena et al. 2008; Su et al. 2012; Lorenzi et al. 2016), subdivided by geographical region and by the existence of non-clonal lineages in areas such as South America, Africa and Asia (Shwab et al. 2014; Galal et al. 2019).

Due to the significant consumption of poultry meat globally, and the potential risk of human infection via this route, elucidating the determinants of spread and risk factors for *T. gondii* infection in poultry is important. In order to monitor the public health risks and the production of safe poultry products in Greece, a seroepidemiological survey of different types of poultry (backyard, organic, and conventional) was conducted to assess the prevalence of *T. gondii* infections and identify potential risk factors for *T. gondii* infection in chickens. *T. gondii* strains were isolated from the tissues and genotyped, and the co-existence of *T. gondii* infections with *Eimeria* spp. was assessed, to elucidate epidemiological links between these two protozoan pathogens.

## Material and methods

### Study design and sampling

**Fig. 1:**
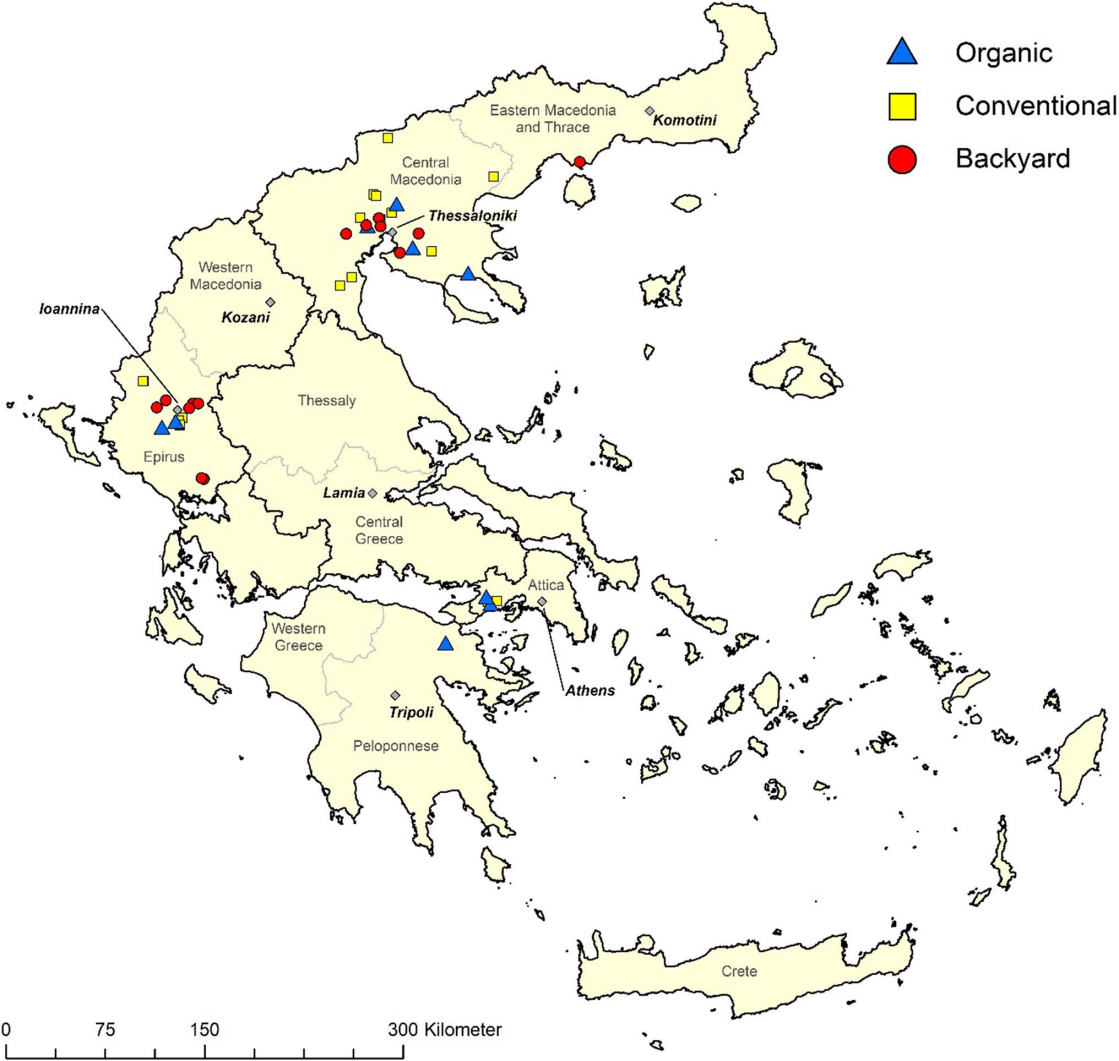
Map of Greece reporting the geolocation of organic (blue triangle), backyard (red circle), and conventional (yellow square) tested farms

The selection of poultry operations was based on the number of commercial farms in three major Greek regions. Poultry farms in Greece are most concentrated in the geographical regions of Epirus (in North-Western Greece), Central Macedonia, and Central Greece-Attica (Hellenic Ministry of Rural Development and Food, http://www.minagric.gr/index.php/en/). For the purpose of the study, sampling from both commercial operations (slow-growing broiler and layer flocks) and backyard farms was conducted proportionately to their frequency and based on sampling convenience (Fig. 1**)**. The study included 42 poultry operations in Greece, sampled between January 2016 and March 2017. Eight of the operations were raising broilers, 14 were backyard farms, and 20 were layer operations. Layer operations were housing chickens either conventionally caged (*n* = 8), conventionally floor housed (*n* = 2), free range (*n* = 2), or as organic flocks (*n* = 8). Backyard operations included housed layer hens of different ages. Broiler flocks sampled for this study were slow-growing chicken or commercial broiler breeds kept under conventional (*n* = 5), free-range (*n* = 2), or organic (*n* = 1) production conditions, based on farm availability. In these flocks, the slaughter age had a 75–100 days range. No intensively-raised broilers were included in this study, as earlier studies have shown very low or even zero prevalence (Dubey et al. 2010b; Matsuo et al. 2014; Rodrigues et al. 2019). In the included broiler flocks, animals were sampled at slaughter, when blood samples and the heart were collected for *T. gondii* detection. In backyard flocks, blood samples were taken from the older animals and in layers from individual animals (at the age of approximately 10 months) according to the expected prevalence. At the time of initial blood sampling, all layer and backyard hens sampled were marked with numbered leg rings on both legs. Depending on producer compliance, flocks were followed up to slaughter where possible in order to re-sample blood and obtain heart tissue during slaughtering. Blood samples (initial and at slaughter) were collected in EDTA tubes, and hearts were placed in individually labelled plastic zipper bags.

At the time of initial blood sampling, faecal samples were also collected from the ground or litter to be tested in a separate investigation for the presence, quantification—expressed in oocysts per gram (OPG) with a modified McMaster technique—and molecular identification of *Eimeria* species by multiple PCRs, as described by Andreopoulou et al. (2022).

In total, 1021 blood samples and 322 hearts were collected from the poultry operations (Table 1). All the blood samples and hearts were transferred immediately to the Parasitology laboratory at the Veterinary Research Institute – Hellenic Agricultural Organization Demeter (Thessaloniki, Greece), where they were stored for further examination. The blood samples were centrifuged to obtain serum and hearts were divided into two sub-samples in order to be processed, as outlined below. They were subject to magnetic capture real-time PCR (mc-PCR) as well as for bioassay in IFNɣ-knockout mice to characterize *T. gondii* isolates. One sub-sample of the hearts and sera were shipped cooled to the Institute of Epidemiology, Friedrich-Loeffler Institute (Greifswald-Insel Riems, Germany) immediately after slaughter, for mouse-bioassay and serologic antibody detection, while the second sub-sample of each heart was shipped to the Institute of Parasitology, Leipzig University (Leipzig, Germany) for *T. gondii* specific mc-PCR. All samples except the heart tissue for bioassay were stored at − 20° C until further analysis.

**Table 1:** Study design and samples collected in total

Farms per region (*n*)	Operation type (*n*)	Total blood samples * collected per operation type (*n*)	Hearts sampled at process (*n*)
Epirus (15)	Layers (4)	107	30
	Slow-growth broilers (4)	141	141
	Backyard (7)	97	0**
Central Macedonia (21)	Layers (10)	296	42
	Slow-growth broilers (4)	82	82
	Backyard (7)	135	27
Central Greece-Attica (6)	Layers (6)	163	0**
TOTAL (42)		1021	322

^*^ Total blood samples in this table refer to both the 1^st^ and 2^nd^ sampling, where available. **No information received about flocks’ processing and slaughter

In the absence of earlier *T. gondii* prevalence data from chickens in Greece, a literature-based estimated prevalence of 10% and 5% was used to calculate the sample size per farm, for backyard, free-range, organic, and conventional flocks, respectively. Precision was set at 10% and confidence level at 95%, and the total number of blood samples collected per flock was calculated based on the flock size using Sampsize (http://sampsize.sourceforge.net/iface) and OpenEpi (version 3.01, http://www.openepi.com/SampleSize/SSPropor.htm).

### Data collection and statistical analysis

Furthermore, a questionnaire was obtained from all included operations in order to acquire additional information for each farm used for the analysis of risk factors associated with *T. gondii* infection. Collected farm-specific information included flock size, farm management and biosecurity, nutrition, outdoor area, presence of cats, rodents, or other animals on the farm premises, production rate and performance, disease history, and poultry health (Supplemental Table 1).

For the identification of potential risk factors, a multilevel modelling (generalized linear mixed modelling fit by maximum likelihood (Laplace approximation)) was performed using R (https://www.r-project.org) version 4.0.2, by applying the package lme4. Because individual animals clustered in farms, “FARMID” was included as a random effect variable. The Akaike information criterion (AIC) was used to characterize the relative model quality, as previously described (Lücht et al. 2019; Basso et al. 2020, Deksne et al. 2022).

After multilevel modelling using the individual explanatory variables (univariable multilevel modelling), a final multivariable multilevel model using more than one explanatory variable was established. Starting from those variables with the lowest AIC in modelling, we added further variables with the lowest AIC during the initial univariable testing. The multivariable multilevel model with the lowest AIC was reported as a final multivariable multilevel model. In all statistical analyses, statistical significance was assumed for *p* values < 0.05.

### TgSAG1 ELISA for chickens

Affinity-purified 30 kDa T*. gondii* surface antigen (TgSAG1, SRS29B; Hosseininejad et al. 2009; Maksimov et al. 2011) was diluted in bicarbonate buffer (0.1 M, pH 8.3) and diluted antigen (30 ng/ml) used to sensitise ELISA plates (120 µl/well, 1 h, 37 °C, PolySorp, Nunc, ThermoFisher Scientific, Denmark). Subsequently, the plates were washed with PBS supplemented with 0.05% (v/v) Tween® 20 (Serva, Heidelberg, Germany) (PBST). A blocking step with 1% casein in PBST (CasPBST; 30 min, 37 °C) followed. The plate was emptied and 100 μl of each serum sample, 1:200 diluted in CasPBST, was added (30 min, 37 °C). After serum incubation, the plates were washed with PBST. Species-specific conjugates (goat anti-chicken IgG (H&L) peroxidase (POD), synonymous to anti-chicken IgY (H&L) (Rockland Immunochemicals; rabbit anti-mouse IgG + IgM (H&L) POD; Dianova, Hamburg, Germany), were diluted 1:4000 in CasPBST. One hundred microliters of diluted conjugate was added to each well and incubated (30 min, 37 °C). Subsequently, the plates were washed with PBST and twice with distilled water. One hundred and fifty µl of 1% tetra-methyl-benzidine (TMB) with 0.012% (v/ v) H_2_O_2_ were added to each well. After 15 min at 37 °C, the reaction was stopped by the addition of 50 µl of 2 M H_2_SO_4_ and the O.D. in each well read at 450 nm. Each sample was tested in duplicate. Positive (PC) and negative control (NC) experimental chicken sera (Maksimov and Schares, unpublished data) or serum of an experimentally infected mouse, and a pre-infection serum (Schares, unpublished data), were tested in quadruplicate on each plate. To normalise ELISA results, ELISA index values (I) were calculated for each sample (S) based on the means of O.D. values: I_S_ = (O.D._S_ — O.D._NC_)/(O. D._PC_ O.D._NC_). A cut-off optimized for maximum diagnostic specificity was applied (ELISA index 0.242) as previously described for the TgSAG1-ELISA_SH_ (Schares et al. 2017).

### Mouse bioassay

Mouse experiments (bioassays) reported in this publication were approved by the Landesamt für Landwirtschaft, Lebensmittelsicherheit und Fischerei of the German Federal State of Mecklenburg-Western Pomerania (Permit 7221.3–2.5–001/13).

For the bioassay, IFNɣ-knockout mice (GKO, IFNɣ -/-, C.129S7(B6)-Ifngtm1Ts/J; animal facility of the FLI, breeding pair purchased from The Jackson Laboratory, USA) were used. The IFNɣ response is essential for resistance against *T. gondii* infection (Schluter et al. 2014). Immunologically impaired mouse strains were therefore used to increase the sensitivity of the bioassay and thus the chance of in vitro isolation of *T. gondii* from mouse tissue. Heart musculature was pepsin-digested, and one mouse was inoculated per hen. Pepsin digestion was performed as described previously (Dubey 1998; Moré et al. 2012). For comparable results, the volumes of the reagents used for digestion, and the final volume after digestion, were strictly based on the weight of the sample. Briefly, per 5 g of ground musculature (without fat and connective tissue), 25 ml of freshly prepared pepsin digestion solution was added (2.6 g of pepsin (1:10,000 biological activity, Sigma Chemical, St. Luis, MO, USA), 5 g of NaCl, 500 ml of sterile distilled water, 7 ml of HCl (25% v/v) to reach a pH 1.1–1.2). Digestion was performed at 37 °C for 1 h, the suspension was filtered through two layers of gauze, and the filtrate was centrifuged at 1200 g for 10 min. The pellet (originating from the ground musculature) was re-suspended in 2 ml of PBS, neutralized by 1.5 ml of 1.2% sodium bicarbonate (pH 8.3), centrifuged at 1200 g for 10 min and the sediment was re-suspended in 1 ml of DMEM that contained 100 IU of penicillin and 100 µg of streptomycin per ml (all volumes given are per 5 g of musculature, initially pepsin-digested). A final volume of 0.5 ml was used for s.c. injection per mouse in the bioassay. Mice were monitored for 42–47 days. If a mouse developed signs of toxoplasmosis (ruffled hair, apathy) during the observation period it was euthanized according to the Federation for Laboratory Animal Science Associations (FELASA) and German regulations, and necropsied.

For strain isolation, the pleural cavity was flushed with 1 ml cell culture medium as well as homogenized brains and lungs of positively tested mice were inoculated on MARC-145 cells as previously described by Schares et al. (2017).

### DNA extraction from pepsin-digested tissues and mouse brains

To extract DNA from mouse tissue and cell cultures, 25 mg aliquots of homogenised mouse brain or 25 µl of cell culture pellets were extracted using the Nucleospin Tissue Kit (Macherey–Nagel, Düren, Germany). To extract DNA from pepsin-digested material, a 200 µl aliquot of the digest was treated with proteinase K as recommended for the Nucleospin Tissue Kit, by scaling up eight times the volumes used for the initial digestion (Macherey–Nagel). After digestion (56 °C, 3 h), 230 µl of the final suspension (1840 µl) was taken and the protocol of the Nucleospin Tissue kit (Macherey–Nagel) was followed as recommended. Approximately, a negative extraction control was represented every tenth sample, which was also tested by real-time PCR identically to the analysed chicken samples.

### Real-time PCR

DNA was analysed by a previously published real-time PCR targeting the 529 bp repeat of *T. gondii* (Talabani et al. 2009), using conditions described previously (Schares et al. 2011; Legnani et al. 2016)*.* A final reaction volume of 20 μl was applied, using a commercial master mix (iQ supermix, Bio-Rad Laboratories GmbH, Munich, Germany) and a CFX96 instrument (Bio-Rad Laboratories). Primers and probes were purchased from MWG-Biotech (Ebersberg, Germany). Real-time PCR primers (800 nM) and a probe (200 nM) were employed as reported (Talabani et al. 2009; Legnani et al. 2016). The cycling conditions were 50 °C for 2 min, followed by 95 °C for 10 min and then 55 amplification cycles of 95 °C for 15 s and 60 °C for 1 min as described (Talabani et al. 2009). After each cycle, the light emission by the fluorophore was measured. Real-time PCR results were analyzed using the CFX manager software Version 1.6 (Bio-Rad Laboratories).

### Genotyping of *T. gondii* isolates

Two methods of *T. gondii* genotyping were applied, one based on PCR–RFLP and the other based on microsatellites. Genotyping of *T. gondii* by PCR–RFLP was performed using eight independent, unlinked chromosomal genetic markers (nSAG2, SAG3, BTUB, GRA6, c22-8, c29-2, L358, PK1) as previously reported (Herrmann et al. 2013) by amplifying DNA extracted from cell culture-derived tachyzoites using internal primers as previously reported (Su et al. 2006).

For microsatellite typing, DNAs extracted from in-vitro isolated tachyzoites were genotyped after multiplex PCR, using 15 markers as described (Ajzenberg et al. 2010). These markers included 8 typing markers (TUB2, W35, TgM-A, B18, B17, M33, IV.1, XI.1) that show little or no variation within lineages and 7 “fingerprinting” markers (M48, M102, N83, N82, AA, N61, N60) that display a high level of polymorphism within clonal lineages type I, type II, or type III (Ajzenberg et al. 2010). The only divergence from the original methods was that in the case of M102, AA, and N60, the fluorophore TAMRA was used instead of NED to label amplicons during multiplex PCR.

### Magnetic-capturePCR (mc-PCR)

The mc-PCR was essentially performed as earlier published (Opsteegh et al. 2010; Schares et al. 2018) with some slight modifications as outlined below.

### Preparation of crude DNA extract

Half of each heart collected (0.9–5 g) was cut using a laboratory grinder or sterile single-use scalpels and then homogenized with the addition of 2.5 volumes lysis buffer (100 mM Tris HCl pH 8.0, 5 mM EDTA pH 8.0, 200 mM NaCl, 0.2% SDS, and 1.2 U/ml of proteinase K) and manual rigorous shaking for approximately 2 min. Samples were placed for overnight digestion in a rocking water bath (85 rpm) at 55 °C and centrifuged for 45 min at 3500 g the next day.

#### Removal of free biotin

Up to 12.0 ml of heart supernatant was incubated at 100 °C for 10 min to inactivate proteinase K. Fifty microliters of triple-washed streptavidin sepharose was added per sample (binding capacity 300 nmol/ml; GE Healthcare, VWR, Germany). Crude extract samples were allowed to cool down below 40 °C followed by streptavidin–biotin binding in a 45-min rotation in an overhead rotator (10 rpm). A new round of centrifugation at 3500 g for 15 min was performed and up to 10 ml of biotin-free supernatant was transferred to clean 15 ml polypropylene tubes.

#### Sequence-specific magnetic capture and qPCR on 529-bp repeat element

The sequence-specific magnetic capture, as well as the real-time PCR were processed as performed by Schares et al. (2018). In short, biotin-labelled capture oligonucleotides Toxo CapF and Toxo CapR (Opsteegh et al. 2010) were added to each biotin-free supernatant, and after DNA denaturation and hybridization of the capture oligonucleotides with *T. gondii* DNA, streptavidin beads were added. After incubation, capture oligonucleotides with hybridised *T. gondii* DNA were isolated by magnet. *T. gondii* DNA was obtained from the bead suspension. The subsequent probe-based real-time PCR amplification was done using the previously described primer–probe combination Tox-SC forward, Tox-SC reverse, and Tox-TP1 (Schares et al. 2018).

The mc**-**PCR results were expressed as Ct values. Results with Ct values < 35 were regarded as positive. Results with Ct values > 40 were regarded as negative. If the Ct value ranged between 35 and 40, the respective amplification curves were visually inspected. If they diverged strongly from those of the positive controls, the samples were regarded as negative.

## Results

### TgSAG1 ELISA on chicken sera

A total number of 934 chickens (backyard, slow-growing broilers, and layers) were serologically examined by the TgSAG1 ELISA. Eighty-eight chickens (9.4%) displayed antibodies against *T. gondii*. More precisely, 73 out of 177 (41.2%) backyard chickens and 15 out of 534 (2.8%) commercial layers were seropositive. All tested slow-growth broiler chickens were seronegative. Of the 15 seropositive commercial layer hens, three were kept in caged layer operations, two in floor-housed conventional operations, four in free-range conventional operations, and six on organic farms (Table 2). A different level of seroprevalence was observed in the three sampling regions, with Epirus recording the highest number of seropositive birds (41/313, 13%), followed by Central Macedonia (44/458, 9.6%). In the Epirus district, a 100% (7/7) backyard flock-level seroprevalence was recorded, while in Central Macedonia it was 85.7% (6/7) (Table 2). As for layers, 50% (1/2) of organic farms in Epirus had seropositive hens, with only 5% (1/20) seropositive birds in this farm and a very low overall regional seroprevalence of 1% (1/104), as estimated for seropositive birds per total birds sampled in this region. Layer farms’ seroprevalence in Central Greece-Attica was 50% (3/6), with 3–5% of birds per seropositive farm having *T. gondii* antibodies and overall regional seroprevalence of 1.8% (3/163), while 60% (6/10) farm seroprevalence was recorded in Central Macedonia with 3 to13% οn-farm seropositivity and a total seroprevalence of 4.1% (11/267) for all birds sampled in that region (Table 3).

**Table 2:** *T. gondii* seropositive poultry operations and range of proportion of on-farm seropositive birds

Operation type (*n*)	Seropositive operations*/*farms sampled per region (%)				Range of on-farm proportion of seropositive birds (%)		
	Epirus	Central Macedonia	Central Greece-Attica	All regions	Epirus	Central Macedonia	Central Greece-Attica
Layers							
- Cages (8)	0/2 (0%)	1/4 (25%)	1/2 (50%)	2/8 (25%)	0%	13%	5%
- Floor (2)	0/0 (NA)	1 (100%)	0 (0%)	1/2 (50%)	NA	9%	0%
- Organic or Free-range (10)	1/2 (50%)	4/5 (80%)	2/3 (66.7%)	7/10 (70%)	5%	3–10%	3%
- Slow-growth broilers (8)	0/4 (0%)	0 /4 (0%)	0/0 (NA)	0/4 (0%)	0%	0%	NA
Backyard (14)	7/7 (100%)	6/7 (85.7%)	0/0 (NA)	13/14 (92.9%)	13–100%	5–88%	NA
TOTAL (42)	8/15 (53.3%)	12/21 (57.1%)	3/6 (50%)	23 (54.8%)	NA	NA	NA

*NA* not applicable

**Table 3:** Seropositive birds per type of poultry, geographical region, and production system

Type of birds (*n*)	Seropositive birds/birds sampled per region (%)			
	Epirus	Central Macedonia	Central Greece-Attica	All regions
Layers (534)				
- Cages (178)	0/58 (0%)	2/80 (2.5%)	1/40 (2.5%)	3/178 (1.7%)
- Floor (40)	0/0 (NA)	2/22 (9%)	0/18 (0%)	2/40 (5%)
- Organic or Free-range (316)	1/46 (2.2%)	7/165 (4.2%)	2/105 (1.9%)	10/316 (3.2%)
- Total layers	1/104 (1%)	11/267 (4.1%)	3/163 (1.8%)	15/535 (2.8%)
Slow-growth broilers (223)	0/141 (0%)	0/82 (0%)	0/0 (NA)	0/223 (0%)
Backyard (177)	40/68 (58.8%)	33/109 (30.3%)	0/0 (NA)	73/177 (41.2%)
TOTAL (934)	41/313 (13%)	44/458 (9.6%)	3/163 (1.8%)	88/934 (9.4%)

*NA* not applicable

### Bioassay of chicken heart tissue from the selected seropositive hens

A total number of 26 hens were selected for bioassay, based on the ELISA results (ELISA index > 0.242) and the farms’ and chickens’ availability. Eleven out of 26 heart samples were tested positive for the presence of *T. gondii* DNA. Bioassay was positive for 7 hens (26.9% of all hens sampled and 63.6% of PCR-positive hens, respectively), all originating from four backyard farms. More specifically, 85.7% of bioassay-positive samples came from backyard farms in Epirus and 14.3% from backyard farms in Macedonia. All seven *T. gondii* isolates belonged to type II, Apico I (ToxoDB#3). Microsatellite typing confirmed that all isolates came from four different farms. All isolates showed farm-specific patterns in microsatellite fingerprinting (Supplemental Table 2), while only within a single farm, identical typing patterns were observed (Supplemental Table 2).

### mc-PCR of chicken heart tissue

Due to impaired sample quality during prolonged tissue shipment, extracted DNA was of sufficient quality and quantity for only 20 out of the 322 sampled hearts. PCR batches were consistently featuring Ct values of around 32 for the 5 × 10^5^ tachyzoites standard and negative controls being negative (Ct > 40). Eight out of the 20 samples were positive (40%) with Ct values of 23–28, indicating a high concentration of *T. gondii* DNA in these samples. Based on that result, a semiquantitative assessment indicates that there was more than 5 × 10^5^ T*. gondii* equivalents in the eight positive samples. Seven positive samples were derived from backyard farms (87.5%) in Central Macedonia. One mc-PCR positive sample originated from a free-range chicken, while in this sampling group 0% seroprevalence was observed. One hen was consistently tested seropositive, mc-PCR positive, and bioassay positive.

### Questionnaire and risk factors analysis

Questionnaire data was obtained from all 42 farms included in the study. Regarding the facility type, 13 farms in total (31%) had concrete floors in the outdoor area, while the remaining 16 (38%) and 13 farms (31%) had grass-soil and bushland, respectively. Nine (56.3%) grass-soil and six (46.2%) bushland outdoor premises belonged to commercial farms. From a management perspective, 22 farms (52%) had drinking lines with nipples, while 20 (48%) farms had cups; however, the latter was mostly seen in backyard farms (13/20, 65%). Out of the 28 commercial farms, 20 (71%) had hanging feeders, while from the remaining eight farms with ground feeders, five (63%) were organic farms. 79% of the commercial farms had automated feeding (22/28). From the 21.4% of the commercial operations that did not, five out of six farms (83.3%) were organic farms. Strikingly, almost all backyard farms (13/14, 92.9%) had ground feeders, with only 42.9% of them partially using ratio in the nutrition of the chickens, relying mainly on free-grazing (57.1%). The presence of cats, application of insecticides or rodenticides, and regular disinfections were reported in 18 (42.9%), 30 (71.4%), and 31 (73.8%) of all farms, respectively.

For the risk factors analysis, no broiler operations were included in the analysis, since they all tested negative for *T. gondii* throughout. The univariable generalized linear mixed modelling revealed an increased risk of *T. gondii*-infection for backyard chickens (*p* < 0.001), while caged layers in conventional systems were not considered a high-risk group. Chickens in flocks of smaller size were found more often *T. gondii* seropositive than others. The outdoor area seems to potentially determine the risk level for *T. gondii* infections; contrary to the presence of concrete floor, it was shown that grass soil (*p* < 0.01) or bushland (*p* < 0.001) significantly increased the infection risk. The presence of other animals within the farm premises, such as rodents, dogs, or sheep, significantly increased the chickens’ risk for *T. gondii* seropositivity (*p* < 0.01). Regular disinfection, application of rodenticides and insecticides, as well as coccidiosis control were putative protective factors (*p* < 0.001, *p* < 0.01, and *p* < 0.01, respectively). The presence of cats alone was not associated with higher *T. gondii* infection risk. Other management features, such as having cups instead of nipples (*p* < 0.001) or round jars (*p* < 0.05) on the ground (*p* < 0.001) as watering and feeding equipment, respectively, were statistically significant putative risk factors to be considered. (Table 4). Overall, animals on *T. gondii* seropositive farms were statistically significantly associated with reports about poor flock performance (*p* < 0.01) and losses in productivity (*p* < 0.05) (Table 4).

**Table 4:** Fixed effects in generalized linear mixed models to determine potential risk factors for *T. gondii* seropositivity in chicken from Greece (univariable multilevel modelling). Data was analysed by bivariable generalized linear mixed modelling including farm identification number (FARMID) as random effects variable in modelling. The Akaike information criterion (AIC) was used to characterize the relative model quality. Only models with statistically significant explanatory variables (*p* < 0.05) or variables tending to be significant (0.05 ≤ *p* < 0.1) are displayed

Model (AIC, model fit)	Variable	Odds ratio (OR)	OR lower 95% CI limit	OR upper 95% CI limit	z-value	*p*	Significance
1 (387.7)	Intercept	0.0672	0.0122	0.37	–3.103	*0.00192*	**
	Region Epirus (ref.)						
	Region Macedonia	0.4104	0.0483	3.48	–0.816	0.41448	
	Region Central Greece-Attica	0.0672	0.0028	1.62	–1.664	0.09607	
2 (355.9)	Intercept	0.75314	0.31701	1.7893	–0.642	0.521	
	Flock Size 1–50 (ref.)						
	Flock Size 51–300	0.01914	0.00324	0.113	–4.367	1.26E-05	***
	Flock Size 301–10,000	0.01269	0.00172	0.0937	–4.282	1.85E-05	***
	Flock Size > 10,000	0.00948	0.00188	0.0478	–5.641	1.69E-08	***
3 (355.1)	Intercept	0.00809	0.00143	0.0457	–5.449	5.07E-08	***
	Production System Cages (ref.)						
	Production System Conv. Floor Indoor	1.06974	0.07531	15.1959	0.05	0.96	
	Production System Conv. Free Range	0.27022	0.01493	4.8902	–0.886	0.376	
	Production System Organic	2.49956	0.31874	19.6013	0.872	0.383	
	Production System Backyard	93.10294	13.58654	637.996	4.617	3.89E-06	***
4 (381.5)	Intercept	2.36E-03	0.000169	0.0331	–4.489	7.16E-06	***
	Housing System Cages (ref.)						
	Housing System Floor Panels	2.56E-09	0	Inf	–0.039	0.9692	
	Housing System Floor Litter	2.63E + 01	1.687215	408.8159	2.333	0.0196	*
5 (374.4)	Intercept	1.32E-03	0.00014	1.25E–02	–5.786	7.22E-09	***
	Outdoor Area Concrete Floor (ref.)						
	Outdoor Area Grass and Soil	3.88E + 01	3.10386	4.86E + 02	2.839	0.004531	**
	Outdoor Area Bushland	1.50E + 02	10.64604	2.11E + 03	3.713	0.000205	***
6 (369.8)	Intercept	0.00463	0.00106	0.0203	–7.137	9.51E-13	***
	Watering Equipment Nipple (ref.)						
	Watering Equipment Cup	47.89544	8.13105	282.1251	4.276	1.90E-05	***
7 (384.0)	Intercept	7.54E-03	0.00142	4.01E–02	–5.732	9.91E-09	***
	Feeding Equipment Linear (ref.)						
	Feeding Equipment Nothing	1.33E + 02	0.53707	3.28E + 04	1.739	0.0821	
	Feeding Equipment Round-Jars	9.45E + 00	1.31405	6.80E + 01	2.231	0.0257	*
8 (370.9)	Intercept	0.00289	5.65E–04	0.0148	–7.018	2.25E-12	***
	Feeding System Hanging (ref.)						
	Feeding System Ground-Hanging	19.07847	7.93E–01	458.918	1.817	0.0692	
	Feeding System Ground	67.07884	1.03E + 01	437.7781	4.395	1.11E-05	***
9 (357.5)	Intercept	0.31145	0.12454	0.779	–2.494	0.0126	*
	Automatic Feed No (ref.)						
	Automatic Feed Yes	0.00957	0.00176	0.052	–5.385	7.23E-08	***
10 (356.1)	Intercept	0.0053	0.00145	1.94E–02	–7.925	2.28E-15	***
	Nutrition Ratio (ref.)						
	Nutrition Free Grazing and Ratio	12.1647	2.5619	5.78E + 01	3.144	0.00167	**
	Nutrition3 Free Grazing	310.7925	49.96315	1.93E + 03	6.154	7.55E-10	***
11 (377.9)	Intercept	0.00187	0.000211	0.0166	–5.636	1.74E-08	***
	Other Animals No (ref.)						
	Other Animals Yes	42.06114	4.136255	427.7154	3.16	0.00158	**
12 (379.1)	Intercept	0.2921	0.05849	1.46	–1.5	0.13368	
	Insecticides or Anti-rodents Drugs No (ref.)						
	Insecticides or Anti-rodents Drugs Yes	0.0426	0.00567	0.32	–3.067	0.00217	**
13 (375.4)	Intercept	0.4572	0.10251	2.039	–1.026	0.304862	
	Disinfections No (ref.)						
	Disinfections Yes	0.0284	0.00442	0.183	–3.751	0.000176	***
14 (376.3)	Intercept	0.0997	0.0328	0.303	–4.064	4.83E-05	***
	Visitors Book No (ref.)						
	Visitors Book Yes	0.0255	0.00306	0.212	–3.393	0.000692	***
15 (382.6)	Intercept	0.0214	0.00658	6.98E–02	–6.376	1.82E-10	***
	Production Losses No (ref.)						
	Production Losses Yes	66.3127	2.18343	2.01E + 03	2.408	0.016	*
16 (380.3)	Intercept	0.1355	0.03543	0.518	–2.921	0.00349	**
	Coccidiosis Control No (ref.)						
	Coccidiosis Control Yes	0.0625	0.00933	0.419	–2.856	0.00429	**
17 (380.9)	Intercept	0.1428	0.04073	0.501	–3.04	0.00237	**
	Coccidiosis Cont. None (ref.)						
	Coccidiosis Cont. Coccidiostatic Rotation	0.0086	0.000447	0.166	–3.151	0.00163	**
	Coccidiosis Cont. Coccidiostatic Shuttle	0.1175	0.001702	8.105	–0.991	0.3215	
	Coccidiosis Cont. Vaccination	0.0528	0.004106	0.679	–2.257	0.02404	*
	Coccidiosis Cont. Other	0.424	0.035264	5.099	–0.676	0.49896	
18 (382.6)	Intercept	0.0195	0.00379	0.101	–4.706	2.52E-06	***
	Poultry Performance Optimal (ref.)						
	Poultry Performance Below Optimal	1.0102	0.13581	7.515	0.01	0.99206	
	Poultry Performance Poor	56.6575	2.7573	1164.206	2.618	0.00885	**
19 (379.6)	Intercept	0.341	0.044	2.643	–1.03	0.3031	
	Number of sheds	0.283	0.1	0.802	–2.375	0.0175	*
20 (383.5)	Intercept	0.0167	0.0016	0.176	–3.411	0.000648	***
	Eimeria McMaster Negative (ref.)						
	Eimeria McMaster Low Intensity	1.6962	0.0787	36.558	0.337	0.735896	
	Eimeria McMaster Medium Intensity	11.3044	0.6311	202.485	1.647	0.099488	
	Eimeria McMaster High Intensity	0.2788	0.0132	5.899	–0.82	0.412062	

Abbreviations: *ref.*, reference; *Inf*., infinite; *CI*, confidence interval; Coccidiosis Cont., Coccidiosis Control; Eimeria McMaster Median Intensities: Low 1–100, Medium > 100–1000, High > 1000;. *p* < 0.1, *p < 0.05, **p < 0.01, ***p < 0.001

In the final multivariable model, free-grazing practices (*p* < 0.05) and the absence of an automated feeding system (*p* < 0.001) appeared to be risk factors for increasing seropositivity (Table 5).

**Table 5:** Fixed effects in a final generalized multivariable linear mixed model to determine potential risk factors for *T. gondii* seropositivity in chicken from Greece (multivariable multilevel modelling). Data was analyzed by bivariable generalized linear mixed modelling including farm identification number (FARMID) as random effects variable in modelling. The Akaike information criterion (AIC) was used to characterize the relative model quality

Model (AIC, model fit)	Variable	Odds ratio (OR)	OR lower 95% CI limit	OR upper 95% CI limit	z-value	*p*	Significance
Final (350.5)	(Intercept)	0.0672	0.0122	0.37	-3.103	0.00192	**
	Nutrition Ratio (ref.)						
	Nutrition Free Grazing and Ratio	1.209	0.13567	10.774	0.17	0.86498	
	Nutrition Free Grazing	17.626	1.48547	209.143	2.274	0.02299	*
	Automatic Feed No (ref.)						
	Automatic Feed Yes	0.0507	0.00555	0.463	-2.642	0.00825	**

No statistical significance was revealed for the seroprevalence differences observed among the three sampling regions, although hens reared in Central Greece-Attica appeared to be protected at a *p* < 0.1 level (Table 4).

### Co-existence with Eimeria spp

On a farm level, *T. gondii* seroprevalence was 54.8% (23/42 farms). Twenty of the *T. gondii*–positive farms showed *Eimeria* spp. oocyst excretion in faecal samples (20/23; 87%). Eight of the combined *T. gondii* and *Eimeria* spp. positive farms were backyard operations (8/20; 40%), seven were organic and free-range layer farms (7/20; 35%) and only one was a conventional floor layer farm (1/20; 5%). Seven of the 20 double-positive farms (63.6%) were positive for more than one intestinal *Eimeria* species. The *Eimeria* spp. observed in *T. gondii* seropositive farms were *E. acervulina* (72.7%), followed by *E. tenella* (54.5%), *E. brunetti* (45.5%), *E. maxima*, and *E. necatrix* (18.2% each), and *E. praecox* (0.09%). Risk factors analysis revealed no statistical association between the intensity of shedding *Eimeria* spp. as expressed in OPG and *T. gondii* seropositivity. However, although statistically significant only at the level of *p* < 0.1, co-infection with *Eimeria* spp. at medium intensity (> 100–1000 OPG) was identified as a risk factor (Table 4).

## Discussion

The aim of the present study was to investigate *T. gondii* seroprevalence and genotypes circulating in chickens in Greece, in both commercial and backyard farms. The overall seroprevalence was 9.5%, which is comparable with chicken studies from other countries (Schares et al. 2017; Tagwireyi et al. 2019; Sarr et al. 2020). We observed significant variations in the proportions of seropositive chickens by poultry type (broilers, commercial layers, backyard poultry). Breaking down the seropositivity results, we observed the highest seroprevalence in backyard chickens (41.2% of backyard birds and 92.9% of backyard farms), which is in accordance with what has been reported for backyard and domestic flocks particularly in Germany (47.7%; Schares et al. 2017), but also Mexico (25.5%; Alvarado-Esquivel et al. 2012), Thailand (64%; Chumpolbanchorn et al. 2009), and Pakistan (17.83% for IgM, 8.8% for IgG; Khan et al. 2020). In layers, a seroprevalence level of 2.8% was identified in the present study. Relatively few studies have been conducted for layers with several of them in China, where a higher prevalence of 7.9% was reported by Xu et al. (2012). Free-range and organic layers had 3.2% seroprevalence, which is similar to the 3.7% seroprevalence reported by Schares et al. (2017) for eastern Germany. All sera collected from slow-growing, free-range, or organic broilers were negative in our investigation, which is different from earlier reports by Rodrigues et al. (2019), although the slaughter age was similar and around 90 days. It seems plausible that prolonged contact with the ground or litter as well as access to the outdoor area including free-grazing practices typically seen in organic and free-range chicken farming, increases the likelihood of *T. gondii* infection. As the demand for slow-growth and/or free-range chicken meat keeps increasing (Ying et al. 2017), special attention should be paid to *T. gondii* infections to reduce potential transmission to humans from undercooked meat or other raw preparations like chicken sausages or carpaccio where there might not be sufficient inactivation of the parasite (Schares et al. 2018). The majority of published studies referring to free-range chickens include both commercial and hobby/backyard farms, with a range of seroprevalence from 6.6% to 90% (Asgari et al. 2009; Cui et al. 2010; Zhao et al. 2012; Moré et al. 2012; Chumpolbanchorn et al.^2013^; Hamidinejat et al. 2014; Gebremedhin et al. 2014; Magalhães et al. 2016; Vismarra et al. 2016; Wang et al. 2016; ElFadaly et al. 2017; Rodrigues et al. 2019; Dubey et al. 2020). This wide range may be due to the use of different serological techniques and cut-off values to detect anti-*T. gondii* antibodies in the chicken sera, including modified agglutination test (MAT), several enzyme-linked immunosorbent assays (ELISA), indirect fluorescent antibody test (IFAT), and indirect haemagglutination (IHA). IHA is usually considered comparatively insensitive (Dubey et al. 2010b) and is linked with a lower reported seroprevalence in the above-mentioned range. In our study, an in-house *T. gondii* surface antigen (TgSAG1, p30, SRS29B) ELISA was used, as earlier established by Schares et al. (2017) to assess *T. gondii* seroprevalence in chickens. The use of *T. gondii* tachyzoite Surface Antigen 1 (TgSAG1) is meant to avoid cross-reactions with other pathogens (Slany et al. 2016; Schares et al. 2018).

Statistically significant seroprevalence differences were not recorded in the three sampling regions at the level of *p* < 0.05 although chickens from Central Greece-Attica tended to be less frequently affected at a level of *p* < 0.1. Prevalence in different geographical areas may be attributed to the sample size, farm housing, and production system, but also to climatic conditions and annual rainfall (Tenter et al. 2000; Dubey et al. 2010b; Mose et al. 2016; Sarr et al. 2020; Khan et al. 2020). The latter parameter is linked with increased soil moisture that can allow oocysts to not only sporulate quicker but also survive longer in the ground (Dubey et al. 2010b). In fact, there are recorded differences in the precipitation among Epirus, Central Greece-Attica, and Central Macedonia. Epirus recorded the highest *T. gondii* seroprevalence (13%), followed by Central Macedonia (9.6%) and Central Greece-Attica (1.8%), with the annual average rain mm being 90.78, 37.04, and 39.43, respectively (http://emy.gr/emy/el/climatology).

For the molecular detection and isolation of *T. gondii* DNA, only heart tissue was used in our study. Several studies have confirmed that the heart is the preferred tissue allowing the detection of viable *T. gondii* stages (Schares et al. 2018; Dubey et al. 2020), which is in line with studies indicating a long-term *T. gondii* DNA persistence in the heart muscle (Geuthner et al. 2014, 2019). Schares et al. (2018) found that slightly fewer samples were positive if tested by mc-PCR compared to bioassay. In our study, only one sample tested positive for both mc-PCR and bioassay. Potentially, this could be related to insufficient DNA extraction in our study that may have led to failure in the detection of parasite DNA; however, some animals tested positive by mc-PCR but not by bioassay. Another factor impacting inconsistencies between *T. gondii* seropositivity and mc-PCR positivity could be the inhomogeneous distribution of the tissue cysts (Hiob et al. 2017). We used only half of the heart per chicken for mc-PCR, which reduced the detection potential. However, despite the possibility of false-negative results, mc-PCR is considered a highly sensitive method that can detect even low parasitic loads, as earlier shown (Hiob et al. 2017; Schares et al. 2018). This was also confirmed in our case, where mc-PCR revealed a positive free-range chicken sample, whereas ELISA was negative at this time, suggesting that a combination of serological and molecular techniques might allow earlier and better monitoring of *T. gondii* infections. We also found four mc-PCR-positive chickens that were bioassay negative. This is in accordance with observations reported by Schares et al. (2018). Interestingly, several chickens were serologically positive both at initial and at slaughter sampling. This hints at a potential long-term presence of antibodies after seroconversion, which might be beneficial for routine testing of chickens used as food animals, especially since overall, seropositivity showed a significant correlation with actual *T. gondii* DNA findings in the chicken heart.

Determination of risk factors associated with *T. gondii* infections in chickens is a widely studied field. Flock size seems to play an important role in other studies. In our study, however, observations were rather determined by the type of farm, production system, and husbandry, as smaller flocks (usually backyard, organic, free-range) seem to correlate with higher *T. gondii* seropositivity rates in chickens. Consequently, backyard and commercial organic farming were revealed as risk factors in our study. These results are in accordance with those of Schares et al. (2017), showing a higher frequency of *T. gondii* infections in small flocks, compared to large free-range layer operations. Cats are considered a very obvious risk factor due to their role as the definitive host of *T. gondii* and were found to correlate with *T. gondii* seropositivity in chickens in several studies (Schares et al. 2017; Vieira et al. 2018a, b; Khan et al. 2020; Chaklu et al. 2020). However, our analysis clearly indicates that the presence of cats alone did not determine the risk or level of seropositivity. Similar findings were reported in many studies where no association between the presence of cats and *T. gondii* seropositivity on a farm level was seen in small ruminants, poultry, and equids (Stelzer et al. 2019). Possible explanations could be either that cats do not easily enter and contaminate commercial farm premises, feed, or water with oocysts or that cats, even if present, may excrete less oocysts if there is a lower prevalence of rodents. This of course depends also on the type of husbandry and management conditions and should be further investigated. On the contrary, other variables, such as the presence of rodents or other animals on the farm (*p* < 0.01), like dogs or sheep, as well as insufficient management, e.g., absence of regular disinfections (*p* < 0.001) or lack of pest control (*p* < 0.01) significantly increase the risk for *T. gondii* infections, further highlighting their role in the dissemination of the parasite. Schares et al. (2017), Vieira et al. (2018a, b), and Chaklu et al. (2020) confirmed the presence of rodents and other animals as factors increasing the risk of infection in chicken farms.

Although chickens have rarely been reported to develop clinical toxoplasmosis (Dubey et al. 2010b) and studies around the economic impact and significance of *T. gondii* infections in poultry are scarce, our risk analysis showed a correlation between *T. gondii* seropositivity and lower reported productivity (*p* < 0.05) or reduced performance (*p* < 0.01). This most likely reflects a more general gap in the sanitary management and biosecurity of the *T. gondii*–positive farms, although more factors might contribute to the impairment of chicken performance. Earlier findings showed that infection of chickens with genotype II *T. gondii* oocysts alone caused no clinical signs (Dubey et al. 1993), but impact of the infection was increased by the presence of other pathogens or diseases (Dubey et al. 2010b). In our study, we had four *T. gondii* seropositive farms, one conventional floor layer farm, and three organic farms, that were positive also for *Eimeria* spp. and reported flock performance below optimal, but more research is needed to check the actual effect of co-existence on flock productivity.

Outdoor access was found to be an important risk factor for *T. gondii* infections, with bushland (*p* < 0.001) and grass or soil (*p* < 0.01) potentially being optimal to maintain the viability of *T. gondii* oocysts. Outdoor access allows chickens to feed on earthworms or insects, that may accumulate oocysts from the environment (Stelzer et al. 2019). Earlier studies by Guo et al. (2015) and Maksimov et al. (2011) showed lower *T. gondii* prevalence in chickens kept strictly indoors, which is in line with our findings. The absence of automated feeding (*p* < 0.01) along with free-grazing (*p* < 0.05) is a combination commonly observed in organic layers and backyard farms in our study. This could contribute to the high level of seropositivity of such birds and the fact that high antibody titers were observed at both blood sampling points. Although age was not included in our analysis, we observed the highest seroprevalence in backyard farms where we sampled the oldest animals. Similarly, Chaklu et al. (2020) found that adult chickens are twice as likely to be seropositive than younger ones, which also aligns with the findings by Khan et al. (2020).

Natural co-infections of chickens with *T. gondii* and *Eimeria* spp. are considered common (Hiob et al. 2017), just as in other animal species (Mason et al. 2015). In our study, *E. acervulina* and *E. tenella* were the species most frequently co-infecting *T. gondii*–positive chickens. Although little knowledge exists on the dynamics and immunological profile of co-infected animals, experimental co-infection with *T. gondii* and *E. tenella* was investigated in vivo (Hiob et al. 2017) and in vitro (Zhang et al. 2018). It appeared that *E. tenella* tended to display increased merogony in *Toxoplasma*-positive birds, although this was not accompanied by more pronounced macroscopic lesions in the gut (Hiob et al. 2017). No synergy was observed in co-infected birds by Dubey et al. (2020). We did not observe any clear association between *Eimeria* spp. OPG levels and *T. gondii* seropositivity, although there was a tendency that a moderate *Eimeria* spp. OPG level was more often found in *T. gondii* seropositive hens. This was not a statistically significant finding but is in line with earlier experimental findings of Hiob et al. (2017) who concluded that *E. tenella* replication might be depressed in chickens orally challenged with *T. gondii* oocysts, although the asexual reproduction of *E. tenella* seemed to be boosted in co-infected birds. However, this assumption needs further investigation. On another note, anticoccidials targeting *Eimeria* spp. might also exhibit anti-*Toxoplasma* activity to some extent. Regarding potential risk factors, the increased co-existence observed in smaller flocks and in production systems that are less restrictive in terms of access to the outdoor area might be biased, since the same factors impact the *T. gondii* mono-infection.

In conclusion, this is the first report of *Toxoplasma* seroprevalence in chickens in Greece, revealing differences per geographical area and production system. Various management practices have been identified as risk factors. Whether there is a mutual interaction in birds co-infected with *Eimeria* spp*.* needs further consideration in experimental studies. Verification of *T. gondii* tissue stages was possible through bioassay and mc-PCR on top of the serological infection confirmation, however, further research is necessary to establish a combined protocol for early, sensitive, and fast detection of *T. gondii* infections in chickens for public health purposes.

## Supplementary Information

Below is the link to the electronic supplementary material.

Supplementary file1 (DOCX 21 KB)

Supplementary file2 (DOCX 20 KB)

## Data Availability

All data generated or analysed during this study are included in this published article.
