# Supplementary material for: Prevalence and molecular characterization of Toxoplasma gondii in different types of poultry in Greece, associated risk factors and co-existence with Eimeria spp
Source: Parasitol Res. 2022 Oct 29;122(1):97–111. doi: 10.1007/s00436-022-07701-6 (PMC9816277; doi:10.1007/s00436-022-07701-6)
Supplement: Supplementary file 1 — Supplementary file1 (DOCX 21 KB) [file 436_2022_7701_MOESM1_ESM.docx]

**Supplemental Table 1:** Questionnaire data classification

| **Data scoring** | **Type of Poultry** | **Region** | **Flock Size** | **Multiage Farm** | **Production System** | **Housing System** | **Outdoor area** | **Warehouse** | **Watering equipment** | **Feeding equipment** | **Automatic feed** | **Collecting eggs** |
| --- | --- | --- | --- | --- | --- | --- | --- | --- | --- | --- | --- | --- |
| **0** |  |  |  | No |  |  |  | No |  |  | No | no |
| **1** | Broilers | Central Macedonia | Jan-50 | Yes | Conventional (cages) | Layers-Floor/litter | concrete floor | Yes | Cup | Ground | Yes | automatically |
| **2** | Layers | Epirus | 51-300 |  | Conventional (floor, indoor only) | Layers-Cages | bush land |  | Nipple | Hanging |  | manually |
| **3** | Breeders | Central Greece | 301-10000 |  | Conventional (free range) | Layers-Floor panels | grass soil |  | Other | Both |  | both |
| **4** | Mixed |  | 10001< |  | Organic | Layers-Combination | other |  |  |  |  |  |
| **5** |  |  |  |  | Backyard | Broilers-Floor/litter |  |  |  |  |  |  |
| **6** |  |  |  |  |  |  |  |  |  |  |  |  |
|  | **Nutrition** | **Cats** | **Rodents** | **Other animals** | **Insecticides/Anti-rodents drugs** | **Disinfections** | **Visitors book** | **Neighbouring Farms** | **Diseases** | **Coccidiosis control** | **Type of Coccidiosis control** | **Poultry Performance** |
| **0** |  | No | No | No | No | No | No | No | no | No |  |  |
| **1** | ratio | Yes | Yes | Yes | Yes | Yes | Yes | Yes | Intestinal | Yes | Coccidiostatic rotation | ok |
| **2** | free-grazing |  |  |  |  |  |  |  | Respiratory |  | Coccidiostatic shuttle | below optimal |
| **3** | both |  |  |  |  |  |  |  | Production losses |  | Vaccination | poor |
| **4** |  |  |  |  |  |  |  |  |  |  | Other |  |
|  | **Toxoplasma serology** | **1st sampling serology result** | **2nd sampling serology result** | **Bioassay result** | **mc-PCR result** |  |  |  |  |  |  |  |
| **0** | negative | negative | negative | negative | negative |  |  |  |  |  |  |  |
| **1** | positive | positive | positive | positive | positive |  |  |  |  |  |  |  |
