# Supplementary material for: Prevalence and molecular characterization of Toxoplasma gondii in different types of poultry in Greece, associated risk factors and co-existence with Eimeria spp
Source: Parasitol Res. 2022 Oct 29;122(1):97–111. doi: 10.1007/s00436-022-07701-6 (PMC9816277; doi:10.1007/s00436-022-07701-6)
Supplement: Supplementary file 2 — Supplementary file2 (DOCX 20 KB) [file 436_2022_7701_MOESM2_ESM.docx]

**Supplemental Table 2.** Microsatellite-typing of parasites isolated from naturally *Toxoplasma gondii* infected chicken sampled from 3 farms using 8 typing and 7 fingerprinting markers, located at various chromosomes.

| **Isolate** |  | **Farm** |  | **Marker (Chromosome)** | | | | | | | | | | | | | | | | **MS type** | |
| --- | --- | --- | --- | --- | --- | --- | --- | --- | --- | --- | --- | --- | --- | --- | --- | --- | --- | --- | --- | --- | --- |
|  |  |  |  | **Typing** | | | | | | | |  | **Fingerprinting** | | | | | | |  | |
|  |  |  |  | **TUB2 (IX)** | **W35 (II)** | **TgM-A (X)** | **B18 (VIIa)** | **B17 (XII)** | **M33 (IV)** | **IV.1 (IV)** | **XI.1 (XI)** |  | **M48 (Ia)** | **M102 (VIIa)** | **N60 (Ib)** | **N82 (XII)** | **AA (VIII)** | **N61 (VIIb)** | **N83 (X)** |  |  |
| RH |  | NA |  | 291 | 248 | 209 | 160 | 342 | 169 | 274 | 358 |  | 209 | 170 | 150 | 119 | 268 | 87 | 306 | I |  |
| ME49 |  | NA |  | 289 | 242 | 207 | 158 | 336 | 169 | 274 | 356 |  | 215 | 176 | 148 | 111 | 268 | 91 | 310 | II |  |
| NED |  | NA |  | 289 | 242 | 205 | 160 | 336 | 165 | 278 | 356 |  | 209 | 192 | 152 | 111 | 270 | 91 | 312 | III |  |
| V84_1 |  | BE1 |  | 289 | 242 | 207 | 158 | 336 | 169 | 274 | 356 |  | 233 | 180 | 149 | 111 | 275 | 85 | 310 | II |  |
| V84_2 |  | BE1 |  | 289 | 242 | 207 | 158 | 336 | 169 | 274 | 356 |  | 233 | 180 | 149 | 111 | 275 | 85 | 310 | II |  |
| V84_4 |  | BE2 |  | 289 | 242 | 207 | 158 | 336 | 169 | 274 | 356 |  | 217 | 180 | 147 | 111 | 265 | 87 | 314 | II |  |
| V84_5 |  | BE2 |  | 289 | 242 | 207 | 158 | 336 | 169 | 274 | 356 |  | 217 | 180 | 147 | 111 | 265 | 87 | 314 | II |  |
| V84_6* |  | BE2 |  | 289 | 242 | 207 | 158 | 336 | 169 | 274 | 356 |  | 217 | 180 | 146 | 111 | 264 | 91 | 314 | II |  |
| V84_8 |  | BE3 |  | 289 | 242 | 207 | 158 | 336 | 169 | 274 | 356 |  | 225 | 180 | 147 | 111 | 265 | 95 | 312 |  |  |
| V86_2 |  | BM4 |  | 289 | 242 | 207 | 158 | 336 | 169 | 274 | 356 |  | 214 | 180 | 144 | 115 | 264 | 103 | 312 | II |  |

* V84_6 was also designated „*T. gondii* Greece” in further studies (Maksimov et al., in preparation)
